# Application of a Social-Ecological Model to Study the Factors Associated With Sarcopenia

**DOI:** 10.7759/cureus.25248

**Published:** 2022-05-23

**Authors:** Junya Shimamoto, Jun Watanabe, Kazuhiko Kotani

**Affiliations:** 1 Division of Community and Family Medicine, Jichi Medical University, Shimotsuke-City, JPN

**Keywords:** social–ecological model, sarcopenia, risk factors, preventative policy, community health

## Abstract

The social-ecological model (SEM) provides a framework for developing multidimensional preventive strategies at the intrapersonal, interpersonal, organizational, community, and policy levels. While sarcopenia forms due to multiple factors, the SEM has not yet been applied to the prevention of sarcopenia. We aimed to categorize the factors associated with sarcopenia in each level of the SEM.

The electronic databases were searched between 2010 and 2021 to collect factors associated with sarcopenia. Information from guidelines, reference lists, and expert consultation was further hand-searched. The factors mentioned in the reviewed studies were classified into the SEM.

In 17 studies, 42 factors associated with sarcopenia were identified; thereafter, 33 biological and individual factors were categorized at the intrapersonal level, isolation was categorized at the interpersonal level, a sedentary lifestyle was at the organizational level, community involvement and social capital were at the community level, as well as knowledge about the disease, disability, income, education, and residential area were categorized at the policy level. The categorization of factors associated with sarcopenia, based on the SEM level, may be useful in the development of preventive strategies; however, further research is required.

## Introduction and background

Sarcopenia is an age-related progressive and generalized skeletal muscle disorder that is caused by multiple factors and was defined by expert panels, such as the European Working Group on Sarcopenia in Older People (EWGSOP) 2 [1] and the Asian Working Group on Sarcopenia [2]. Among community dwellers over 60 years of age, the prevalence of sarcopenia is estimated to be approximately 10% in both men and women [3]. Risk factors, such as aging, physical inactivity, and poor nutrition, are well known, and most factors have biological and individual backgrounds [1,2]. Simultaneously, socioeconomic factors are also involved in the cause and effect of sarcopenia [1,2]. A multidisciplinary approach is thus required to prevent sarcopenia; thus, it would be of paramount importance to display the factors associated with sarcopenia from multiple viewpoints [4].

The social-ecological model (SEM) is a schematic framework designed to improve our understanding of disease at multiple levels (i.e., intrapersonal/individual, interpersonal, organizational/institutional, community, and system/public policy levels) [5]. In addition, the SEM indicates the interactive characteristics of individuals and environments to facilitate the development of multidisciplinary preventive strategies in public health organizations (e.g., the National Institutes of Health and Centers for Disease Control and Prevention) [6]. Indeed, the SEM has been used in fields such as suicide, violence, childhood obesity, influenza vaccination, and human immunodeficiency virus and cancer screening [6].

Given the multifactorial manifestations of sarcopenia, there seems to be a need to address risk factors related to the prevention of sarcopenia using the SEM. The purpose of our present work is to outline the factors associated with sarcopenia and categorize the factors into each level of the SEM, which has been never performed. This would be useful for understanding sarcopenia and its risks, as well as for promoting prevention strategies of sarcopenia based on the level of SEM.

## Review

Materials and methods

The present systematic review adhered to the guidelines of the PRISMA-ScR (Preferred Reporting Items for Systematic reviews and Meta-Analyses extension for Scoping Reviews) checklist for the reporting of findings [7]. To strengthen the rigor of our scoping review, we used a scoping Arksey and O’Malley framework [7], which consists of the following steps: identify the research question and relevant studies, select studies, chart the data, and collate/summarize.

Identify the Research Question and Relevant Studies

The goal of this literature review was to identify and categorize the factors associated with sarcopenia at each level (i.e., intrapersonal, interpersonal, organizational, community, and policy levels) in the SEM (Table 1) [5]. The following search terms were used: “sarcopenia”[Mesh] OR “Muscle Weakness”[Mesh] OR “Muscular Atrophy”[Mesh] for factors. The search was conducted in four electronic databases: PubMed (research in medical interventions), Cochrane Central Register of Controlled Trials, Embase, and Cumulative Index to Nursing and Allied Health Literature (CINAHL). In consideration of the possibility that information was not fully collected by the above methods, relevant studies were further hand-searched from international guidelines [1,2], reference lists of the reviewed articles, and consultation with experts in the field of sarcopenia.

**Table 1:** A level of an ecological perspective

Level	Definition
Intrapersonal	Individual characteristics that influence behaviors, such as knowledge, attitudes, beliefs, and personality traits
Interpersonal	Interpersonal processes and primary groups, including families, friends, and peers that provide social identity, support, and role definition
Organizational	Rules, regulations, policies, and informal structures that may constrain or promote recommended behaviors
Community	Social networks and norms, or standards, which exist formally or informally among individuals, groups, and organizations
Policy	Policies and laws that regulate or support healthy actions and practices for disease prevention, early detection, control, and management

Select Studies and Chart the Data

The following inclusion criteria were applied: 1) availability of full text; and 2) publication between January 2010 and June 2021. To use the international guideline definition of sarcopenia, studies before 2010 were excluded. Two reviewers (JS and JW) independently conducted screening and further reviews. First, the title and abstracts were compared against the inclusion criteria. The articles that appeared to meet the criteria and those for which there was any uncertainty were obtained as full-text articles. Then, both reviewers screened the full text for inclusion. To confirm the robustness, the eligibility of the collected articles was discussed between the two reviewers until a consensus had been reached. Arbitration was conducted by a third reviewer (KK) if required. Two reviewers independently extracted the data from the included studies. We extracted key information about the selected articles (i.e., authors, publication year, country, findings). If data on participants, interventions, or study characteristics were missing or not sufficiently described, we contacted the corresponding authors and tried to obtain missing information.

Collate/Summarize

Findings regarding factors associated with sarcopenia were aggregated, and the authors classified the factors into the level of the SEM [5]. Concrete categorization was conducted with the following thoughts: the intrapersonal level was based on the biological and individual properties. Factors based on various networks (e.g., family and friends) were included at the interpersonal level. Factors at the organizational level included overall behaviors in relation to regulations and informal structures. Factors associated with social connection and cultural context were included at the community level. Factors necessary for system/public initiatives were included at the policy level. Any discrepancies were resolved through in-depth discussion with the involvement of the third review author (KK) to achieve consensus.

Results

Study Selection

**Figure 1:**
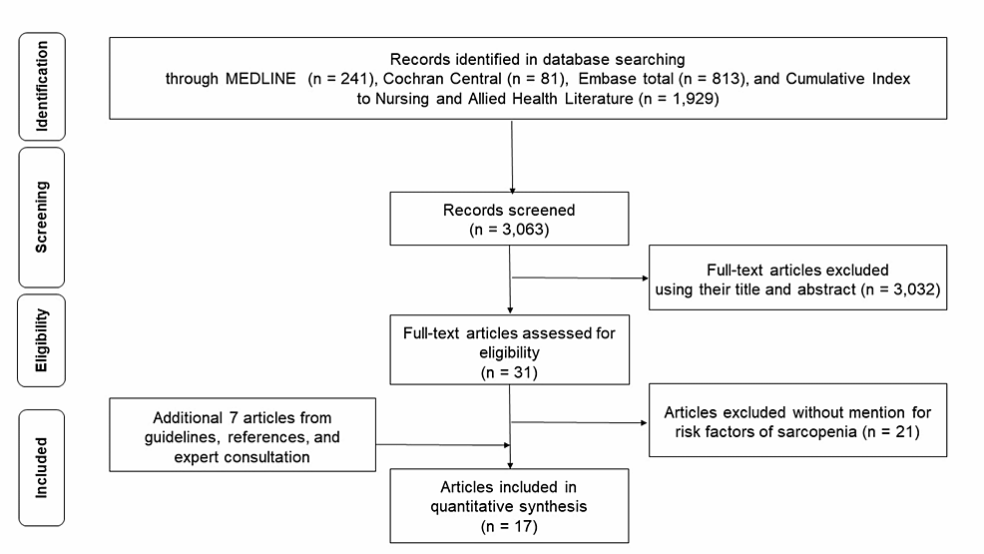
Process of literature search with Preferred Reporting Items for Systematic Reviews and Meta-Analyses flow diagram.

Figure 1 shows the flow chart of the article search. A total of 3,063 papers were identified in PubMed (n = 241), the Cochrane Central Register (n = 81), Embase (n = 813) and CINAHL (n = 1,929). In the first screening, 3,032 articles were excluded based on a review of the title and abstract. In the second full-text screening of 31 studies, we excluded 21 studies that did not exactly mention the factors. Seven studies were collected from references in the searched studies and consultation with experts. As a result, we identified 17 studies [8-24] for the present study.

Descriptive Findings From the Reviewed Papers

**Figure 2:**
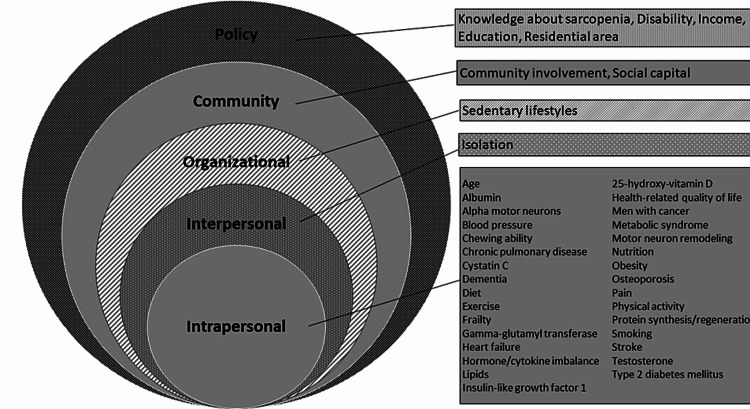
Categories of identified factors for sarcopenia based on the social-ecological model.

Table 2 summarizes the profiles of the included studies. The included studies were published from 2015 to 2021. These studies were conducted in North America, the Middle East, Latin America, Europe, Oceania, and Asia. Based on Table 2, Figure 2 shows the categorization of factors associated with sarcopenia. Overall, 43 factors were identified and the following factors were repeatedly mentioned across the studies: physical inactivity (n = 9), poor diet (n = 3), dementia (n = 2), obesity (n = 2), heart disease (n = 2), chewing disability (n = 2), chronic obstructive pulmonary disease (COPD) (n = 3), renal failure (n = 2), osteoporosis (n = 2), muscle motor impairment (n = 2), hormone imbalance (n = 2), smoking (n = 2), and social capital (n = 2). Here, we differentiated physical activity/exercise at the intrapersonal level from sedentary life as an overall behavioral pattern (for reference to the Global Action Plan on Physical Activity 2018-2030 by the World Health Organization) [25]. The biological determinants of health, as well as individual pathological traits, were categorized into the factors at the intrapersonal level. This included factors expressed by laboratory biomarkers (e.g., abnormal levels of albumin, cystatin C, gamma-glutamyl transferase, and 25-hydroxy-vitamin D, metabolic abnormalities, motor neuron remodeling, protein synthesis and regeneration, testosterone, insulin-like growth factor 1, alpha motor neuron, hormone balance, and cytokine balance). Isolation (i.e., living alone) was considered a factor at an interpersonal level [21]. A sedentary lifestyle was classified as an organizational factor [22].

**Table 2:** Profiles of studies reviewed

Authors [ref no.]	Year	Country	Factors of sarcopenia mentioned (to be improved)
Kimura [8]	2013	Japan	Disability, community involvement
Filippin [9]	2015	Brazil	Physical activity, metabolic syndrome, cognitive function
Steffl [10]	2015	Prague	Smoking
Giallauria [11]	2016	Italy	Physical activity, muscle use, alpha motor neuron
Imamura [12]	2016	Japan	Social capital
Kim [13]	2016	Japan	Age, body mass index, overweight, chewing ability, 25-hydroxy-vitamin D, insulin-like growth factor 1, testosterone, albumin, gamma-glutamyl transferase, lipids, cystatin C, blood pressure, osteoporosis, pain, stroke, chronic kidney disease, chronic obstructive pulmonary disease, fat and protein intake, exercise, instrumental activities of daily living
Dennison [14]	2017	UK	Men with cancer, diet, physical activity, smoking, obesity
Reijnierse [15]	2017	Netherlands	Knowledge about sarcopenia, equipment availability
Vlietstra [16]	2018	New Zealand	Physical activity, frailty, heart failure, chronic obstructive pulmonary disease, renal failure, osteoporosis, obesity, dementia, Type 2 diabetes mellitus, health-related quality of life
Mundi [17]	2018	USA	Dietary protein
Shen [18]	2018	China	Nutrition
Coelho-Junior [19]	2020	Italy	Income
Dae-Woo [20]	2020	Korea	Chewing ability
Nguyen [21]	2020	Viet Nam	Age, body mass index, chronic obstructive pulmonary disease, male, malnutrition, physical activity, community life, isolation
Wahlen [22]	2020	Qatar	Sedentary lifestyles, hormone and cytokine balance, protein synthesis and regeneration, motor neuron remodeling, early developmental inﬂuences, chronic heart failure
Gandham [23]	2021	Australia	Physical activity, education, residential area
Zidroul [24]	2021	Greece	Social capital

Discussion

We identified 42 factors associated with sarcopenia. Then, based on the SEM, 33 of these factors were categorized at the intrapersonal level, isolation was categorized at the interpersonal level, sedentary lifestyle was categorized at the organizational level, community involvement and social capital were categorized at the community level, and knowledge about the disease, disability, income, education, and residential area were categorized at the policy level. The findings obtained by displaying the factors associated with sarcopenia at each level of the SEM would be of value to understanding sarcopenia and its risk factors at multifaceted levels and for promoting interactive approaches to the prevention of sarcopenia.

In the present work, many factors were found at the intrapersonal level and several factors were found at the other levels. This might partly reflect that biological and individual research has been mainly conducted to clarify the risk factors for sarcopenia, as it is in other medical fields in general. The prevention of sarcopenia is not always thought to be sufficient to exert influence on individuals and their surrounding environments, given the multifactorial manifestations of sarcopenia [1,2,4]. In other words, the observation that the present work found many factors at the intrapersonal level may encourage more studies at other levels.

The approach to the factors categorized at the intrapersonal level is easily understood; that is, lifestyle modification and medical treatment are usually provided to individuals in health services and clinics. The approach to environments may be hard to be understood directly, but some examples exist. For example, at a familial interindividual level, if a family member takes care to ensure that protein-containing food is available in daily life and this food intake is shared in a family, it may naturally contribute to the prevention of sarcopenia in all members as an interpersonal-level approach. Workplace health promotion can help individuals in the same workplace to adopt and maintain healthy lifestyle behaviors [25]. As sedentary lifestyle does not simply mean exercise, promotion of use of stairs instead of elevators as an organizational approach can help improve the sedentary lifestyle [26]. An exercise program in the local community, as a community-level approach, will enable individuals to reduce their risk of type 2 diabetes mellitus [25]. The system of annual health checkups that include community-based screening for diabetes mellitus, a cause of sarcopenia, can lead to the prevention of sarcopenia [27]. Community-based care services following checkups for sarcopenia and frailty are provided in the local community [28]. Social policies for improving income, education, disability, and residential areas may enable more effective prevention of sarcopenia as policy-level approaches [8,18,22]. Although these examples do not appear to be planned with an understanding of SEM-based categorization at present, more approaches will be discussed if each approach is sorted based on the SEM.

The present scoping review was associated with several limitations. First, the search methods (e.g., with respect to search terms used) may have been restrictive for collecting information, although comprehensive information is needed to use the SEM. We made an effort to use standard methods for the review, and we completed a full inspection of references in the included studies and collected additional information from experts. Second, the definition of sarcopenia was different depending on the year of publication because the new criteria reported by the EWGSOP in 2018 allow for the diagnosis of probable sarcopenia based on grip weakness alone [1]. However, because “muscle weakness” was included as a search keyword, we could search for studies similar to the EWGSOP criteria before 2018. Third, the present review was not conducted with the aim of evaluating the quality of the studies that were analyzed. The summary of the review was based on the fundamental evidence of the studies rather than their intrinsic quality. Nevertheless, we think that our first use of the SEM in the development of preventive strategies against sarcopenia would be of value for further advancing this topic.

## Conclusions

In summary, the present study using the SEM sorted the biological and individual factors of sarcopenia at the intrapersonal level and other factors at the interpersonal level (isolation), organizational level (sedentary lifestyle), community level (community involvement and social capital), and policy levels (knowledge about the disease, disability, income, education, and residential area). The SEM-based categorization of factors associated with sarcopenia may serve to improve the understanding of sarcopenia and its risks as well as interactive approaches to prevent sarcopenia, and in turn, be applied in the development of multidimensional strategies against sarcopenia. Further confirmation of the categorization and implementation of approaches based on the categorization is required.
